# Introduction to Special Issue: Visual Mental Imagery System: How We Image the World

**DOI:** 10.3390/vision9030059

**Published:** 2025-07-14

**Authors:** David F. Marks

**Affiliations:** Independent Researcher, 13200 Arles, Provence-Alpes-Côte d’Azur, France; dfmarksphd@gmail.com

The aim of this Special Issue is to provide new perspectives on the role of visual mental imagery in how we image the world, past, present and future. This Special Issue comprises research articles from across the spectrum of relevant empirical sciences. We are interested in the role of visual and mental imagery in cognition, imagination, and action directed towards the present or anticipated states of the external world. This Special Issue was designed to enhance our understanding of how human beings image the world with self-awareness in relation to a challenging and changing external environment. Within this theme, there was scope for studies addressing individual differences, phenomenology, imagination, creativity, artistic expression, forecasting, and futures studies. This Special Issue consists of ten articles.

The first article (Contribution 1), by David F Marks, reviews phenomenological studies in visual–mental imagery (VMI) over early (1860–1929), middle (1930–1999), and recent (2000–2023) time periods. The review focuses on two forms of constructive VMI—memory and eidetic imagery—that vary along a continuum of vividness. Vividness is defined as “a combination of clarity, colourfulness and liveliness, where clarity is defined by brightness and sharpness, colourfulness by image saturation and liveliness by vivacity, animation, feeling, solidity, projection and metamorphosis.” The author integrates his findings into a template with 16 vividness properties to reveal a high degree of consistency in the reported VMI characteristics across time, space, and cultures.

The second article, by Nadine Dijkstra (Contribution 2), addresses the question of whether the early visual cortex (EVC) is necessarily involved in VMI. Dijkstra proposes that inconsistencies in findings regarding the EVC in VMI can be understood as being due to “unique challenges associated with investigating EVC activity during imagery.” The author suggests that EVC activity relates to specific imagery properties. Methods sensitive to variation in low-level features indicate that imagery is able to recruit the EVC in similar ways to those of perception. However, if the requisite level of detail is absent, then the EVC is unnecessary in VMI.

The third article, by Feiyang Jin, Shen-Mou Hsu, and Yu Li (Contribution 3), comprises a systematic review of search of English-language articles published between 2015 and the present. The authors pinpoint three primary areas of disagreement: whether aphantasics exhibit impairments solely in visual imagery, whether a distinction should be made between the absence of voluntary and involuntary imagery, and whether aphantasia primarily concerns the generation of imagery. The authors provide a cutting-edge review of the conceptual and methodological issues at the core of aphantasia research. They suggest that future research is needed to “refine the definition and diagnosis of aphantasia, strengthen empirical investigations at behavioral and neural levels, and, more importantly, develop or update theories”.

The fourth article, by Andrea Blomkvist (Contribution 4), reviews the contributions of the hippocampus to mental imagery formation, especially evidence supporting the view that the hippocampus contributes to the spatial model used for mental imagery. The author argues that different hippocampal circuits contribute to different types of imagery, such as object imagery, scene imagery, and imagery with a temporal aspect. Blomkvist suggests that new avenues should be opened up for research into the role of the hippocampus across a variety of imagery tasks.

The fifth article, by Takao Hatakeyama (Contribution 5), examines empirical associations between imagination, mental imagery, and relevant cognitive abilities and autistic traits. The author hypothesizes that imaginative impairments and distinctive sensory characteristics are to be expected in the mental imagery capabilities of individuals with autism spectrum disorder (ASD). Two vividness tests and one imagery-type test were used to assess imagery ability in 250 college students. A high-scoring ASD group exhibited lower visualization scores, indicating that autism-like traits are associated with mental imagery ability.

The sixth article, by Hiroki Motoyama and Shinsuke Hishitani (Contribution 6), presents a study of the brain regions associated with the perception-driven suppression of mental imagery generation. The focus of the article is the left posterior cingulate gyrus (PCgG). The authors compared brain activation in a picture observation condition and a positive imagery generation condition. Significantly higher activation occurred in the left PCgG in the former condition compared to the latter condition. A significant correlation was observed between the activation of the left PCgG and participants’ subjective imagery richness ratings, which are a measure of the clarity of a presented picture. The results suggest that the left PCgG plays a role in suppressing the generation of mental imagery.

The seventh article, by Mohamad El Haj and Ahmed A. Moustafa (Contribution 7), investigates eye movements during near- and distant-future thinking. Previous research suggests that near-future events are typically viewed from a first-person (own-eyes) perspective while distant-future events are typically viewed from a third-person (observer) perspective. Would these distinct mental perspectives be accompanied by distinct eye movement activities? To test this hypothesis, El Haj and Moustafa invited participants to imagine near- and distant-future events while recording their eye movements with eye-tracking glasses. Fewer, longer fixations were observed for near-future thinking than for distant-future thinking, and more “field” mental visual perspective responses were evident for near-future thinking than for distant-future thinking. This study demonstrates the links between the temporality of future thinking triggers and distinctive mental imagery and eye movement patterns.

The eighth article, by Ekaterina Pechenkova, Mary Rachinskaya, Varvara Vasilenko, Olesya Blazhenkova, and Elena Mershina (Contribution 8), reports an investigation into the ability to adopt different perspectives in mental imagery. Pechenkova et al. used fMRI to compare functional connectivity during first-person and third-person perspective taking among 26 participants performing two spatial egocentric imagery tasks, imaginary tennis and house navigation. Although no significant differences were indicated, a “small subnetwork of early visual and posterior temporal areas” yielded stronger functional connectivity during the first-person perspective, suggesting the possibility of an associated “loop between long-term memory and the “visual buffer” circuits.”

The ninth article, by Maren Bilzer and Merlin Monzel (Contribution 9), describes a study comparing the multisensory profiles of voluntary mental imagery and dream imagery in 226 participants. Bilzer and Monzel report an association between the vividness of voluntary mental imagery and the vividness of dream imagery that appeared to be moderated by the frequency of dream recall and lucid dreaming. Emotional and visual imagery were significantly more vivid for dream imagery than for waking imagery, while the vividness of auditory, olfactory, gustatory, and tactile imagery was higher for waking mental imagery.

The tenth article (Contribution 10), by David F Marks, reports a new analysis of the association between visual short-term memory (VSTM) and the brain-regional volumes of the hippocampi, primary visual cortices, and other cortical regions among vivid and non-vivid visual imagers. In a sample of 53 volunteers aged 54 to 80 with MRI scans, the performance of 10 Low VVIQ scorers and 10 High VVIQ scorers was compared. The subject groups included an aphantasic person and a hyperphantasic person. The High-VVIQ group yielded (i) significantly more accurate VSTM performance and (ii) significantly larger volumes of the hippocampi and primary visual cortex. For 47 subfields, the volumes in the hyperphantasic person exceeded those in the aphantasic person by an average of 57 percent. The study confirms the existence of robust empirical associations between VMI vividness, short-term memory, the regional volume of hippocampal subfields, and V1 area.

Coda

The aim of the Special Issue is to provide new perspectives on the role of visual mental imagery in how we image the world, both present and future. This network of international researchers has demonstrated a vital, dynamic, and flexible role for VMI in mental life. Three primary functions of VMI include (A) remembering recent- and distant-past *stimuli*, *episodes*, *events and scenarios* (“SEES”); (B) anticipating, foreseeing, and simulating near- and distant-future SEES; and (C) constructing phantasy for dreams and imaginary SEES. A key issue to investigate in future research is how, in rare cases (in this Guest Editor’s opinion), voluntary VMI can be absent, and to what extent VMI absence could be effectively compensated for.

We look forward to further exciting discoveries that will help us to understand how we image the world.

